# *O*-acetylation of typhoid capsular polysaccharide confers polysaccharide rigidity and immunodominance by masking additional epitopes

**DOI:** 10.1016/j.vaccine.2019.05.050

**Published:** 2019-06-27

**Authors:** Krisztina Hitri, Michelle M. Kuttel, Gianluigi De Benedetto, Kay Lockyer, Fang Gao, Peter Hansal, Timothy R. Rudd, Emma Beamish, Sjoerd Rijpkema, Neil Ravenscroft, Barbara Bolgiano

**Affiliations:** a Division of Bacteriology, National Institute for Biological Standards and Control (NIBSC), Blanche Lane, South Mimms, Potters Bar, Hertfordshire EN6 3QG, UK; b Department of Computer Science, University of Cape Town, Private Bag X3, Rondebosch, 7701 Cape Town, South Africa; c Laboratory of Molecular Structure, Analytical and Biological Sciences, NIBSC, Blanche Lane, South Mimms, Potters Bar, Hertfordshire EN6 3QG, UK; d Department of Chemistry, University of Cape Town, Private Bag X3, Rondebosch, 7701 Cape Town, South Africa

**Keywords:** Enteric, Glycoconjugate, Molecular modelling, Nuclear magnetic resonance, Vaccine, Vi polysaccharide, HPAEC-PAD, high-performance anion-exchange chromatography coupled with pulsed amperometric detection, HSQC, heteronuclear single quantum coherence spectroscopy, MD, molecular dynamics, mAb, monoclonal antibody, Mw, weight-average molecular mass, SEC-MALS-RI-Viscometry, Size-exclusion chromatography coupled with multi-angle light scattering, refractive index, and viscometry, Vi, Vi polysaccharide

## Abstract

In this work, we explore the effects of *O*-acetylation on the physical and immunological characteristics of the WHO International Standards of Vi polysaccharide (Vi) from both *Citrobacter freundii* and *Salmonella enterica* serovar Typhi. We find that, although structurally identical according to NMR, the two Vi standards have differences with respect to susceptibility to de-*O*-acetylation and viscosity in water. Vi standards from both species have equivalent mass and *O*-acetylation-dependent binding to a mouse monoclonal antibody and to anti-Vi polyclonal antisera, including the WHO International Standard for human anti-typhoid capsular Vi PS IgG. This study also confirms that human anti-Vi sera binds to completely de-*O*-acetylated Vi. Molecular dynamics simulations provide conformational rationales for the known effect of de-*O*-acetylation both on the viscosity and antigenicity of the Vi, demonstrating that de-*O*-acetylation has a very marked effect on the conformation and dynamic behavior of the Vi, changing the capsular polysaccharide from a rigid helix into a more flexible coil, as well as enhancing the strong interaction of the polysaccharide with sodium ions. Partial de-*O*-acetylation of Vi revealed hidden epitopes that were recognized by human and sheep anti-Vi PS immune sera. These findings have significance for the manufacture and evaluation of Vi vaccines.

## Highlights

• The binding of anti-Vi mAb and polyclonal immune sera correlated with the level of *O*-acetylation.
• *C. freundii* Vi resists de-*O*-acetylation and is more viscous than *S.* Typhi Vi.
• Sera from human vaccine recipients contains IgG that recognizes the backbone of Vi.
• Simulations show *O*-acetyls are exposed on the surface of Vi and confer rigidity.
• MD gives conformational rationale for effect of *O*-acetylation on Vi antigenicity and viscosity.

## 1 Introduction

**Fig. 1:**
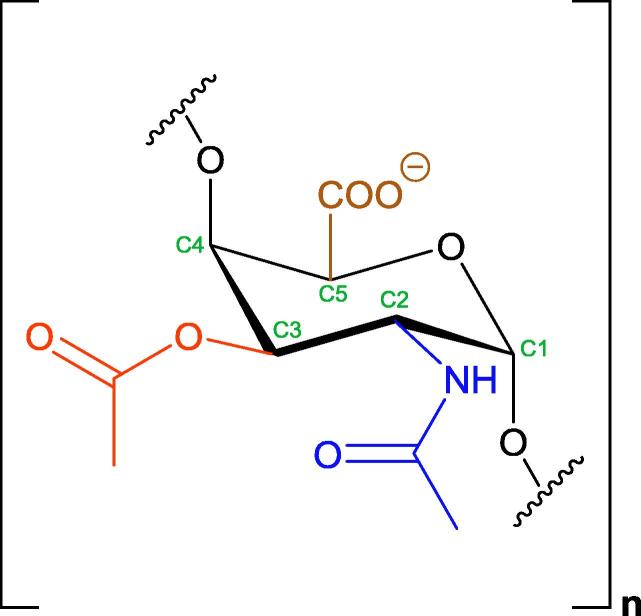
Chemical structure of Vi polysaccharide. Vi is a homopolymer of repeating units of α(1 → 4)-*N*-acetylated-D-galactosaminuronic acid (GalNAcA), *O*-acetylated at the C-3 position. The color scheme is the same as used in Fig. 7, Fig. 8: ring carbons (), *N*-acetyl (), *O*-acetyl (), and carboxyl ().

*Salmonella enterica* subspecies *enterica* serovar Typhi (*S.* Typhi) causes typhoid fever in humans and is protected from host defenses by its capsular polysaccharide (PS), Vi (a *Vi*rulence Antigen) [1], [2]. Vi PS (referred to herein as Vi) is a linear homopolymer of poly-α(1 → 4)-*N*-acetylated -D-galactosaminuronic acid (GalNAcA), *O*-acetylated at the C-3 position (Fig. 1) [3], [4]. A sufficient level of Vi *O*-acetylation in parenteral vaccines has long been recognized as essential for conferring protection against enteric disease, and guidelines recommend the equivalent of >52% *O*-acetylation [2], [5], [6], [7], [8]. Immunogenicity studies in mammals, including humans, have confirmed that the *O*-acetyl group is the dominant epitope of Vi [9], [10]. Partial or complete removal of *O*-acetyl groups from the PS backbone results in reduced antigenicity [9], [10], [11], [12] and immunogenicity in clinical trials [2], [13]. Therefore, for Vi and Vi-protein conjugate vaccines, the level of *O*-acetylation is regarded as a measure of vaccine potency [7], [8]. WHO International Standards (ISs) for Vi polysaccharides from *S.* Typhi and *Citrobacter freundii*, which produce structurally identical PS [14], [15], have been established as quantitative standards for measuring the saccharide content of vaccines using a variety of physicochemical and immunological assays [16].

The impending licensure of Vi conjugate vaccines necessitated the development of physicochemical and immunochemical assays to quantify free and conjugated Vi. Vi behaves differently to other anionic bacterial capsular polysaccharides used in conjugate vaccines, with poor matrix interactions and low recovery from columns, especially for the Vi from *S.* Typhi [17]. Poor solubility and low yields during manufacture have been attributed to the *O*-acetylated surface of Vi. A WHO collaborative study for the quantitation of Vi in vaccines concluded that the potency of *S.* Typhi Vi vaccine is more accurately determined using the homologous Vi as standard rather than Vi from *C. freundii*, inferring a difference between the two Vi standards [16]. Further characterization of these standards to better understand their behavior in solution was therefore warranted.

We aim to characterize the hydrodynamic behavior of both Vi standards using meningococcal PSs as comparators, for size (molar mass and hydrodynamic radius) and viscosity measurements. A secondary aim is to investigate the immunogenicity of the *O*-acetyl group by probing the contribution of *O*-acetylation to the post-immunization anti-Vi IgG response across a panel of PS with a range of degrees of de-*O*-acetylation. To elucidate the structural consequences of de-*O*-acetylation, we employ NMR measurements and molecular dynamics simulations (MD) to probe the effect of *O-*acetylation on the conformation and dynamics of the Vi polymer [18].

## 2 Materials and methods

### 2.1 Materials

WHO ISs for *C. freundii* Vi (NIBSC code 12/244), *S.* Typhi Vi (16/126) [16], *Neisseria meningitidis* group A PS (MenA PS, 13/246), MenC PS (08/214), MenW PS (16/152), MenY PS (16/206), and MenX PS (14/156) were analyzed [19], [20] (see https://www.nibsc.org for product information). Polyclonal anti-Vi IgG included two human sera both prepared by immunization with Vi-TT conjugate vaccine: the WHO 1st IS for anti-typhoid capsular Vi IgG, human (16/138 [21], [22]), and an anti-Vi IgG (10/126 [21]); and, a sheep hyperimmune serum (07/160). Sheep anti-Vi serum 07/160 was prepared by four booster immunizations with one single human dose of Vi-rEPA (recombinant *Pseudomonas aeruginosa* exotoxin A) conjugate vaccine [23] in Freund’s adjuvant. A mouse monoclonal IgG1 Kappa anti-*S.* Typhi Vi (Oxford Biosystems) was also used. Freeze-dried standards were stored at −20 °C and Milli-Q deionized water was used for their reconstitution to ≥1 mg/mL. Further information about the antisera and Ab reagents is given in Supplementary Table S1.

### 2.2 Sample preparation

De-*O*-acetylation of Vi was by base treatment with ammonium hydroxide (NH_4_OH, Sigma, 221228-M). Vi was dissolved in 500 to 2000 µL of Milli-Q water to a concentration of 1 to 4 mg/mL. The solutions were transferred in 500 µL volumes to glass screw cap vials. Water or 10 M NH_4_OH was added to give 0.5 mg/mL to 1.9 mg/mL Vi and 0.05 M, 0.3 M, 0.7 M, 1.0 M and 1.5 M NH_4_OH, in duplicate. NH_4_OH was added by hand, drop-by-drop, to avoid denaturation. The solutions were incubated at 37 °C for 18 h. Desalting to remove free acetyl groups and NH_4_OH was by dialysis using 10 kDa MWCO Spectrapor 10 membranes (Spectrum Laboratories) with three exchanges using 0.85% to 0.085% w/v NaCl solutions, and 3 changes against water. Subsequently, centrifugal evaporation by SpeedVac (Eppendorf) was performed for 10 h, and the pellet was reconstituted in 1.0 mL of water to 1.94 and 2.03 mg/mL *C. freundii* or *S.* Typhi Vi, respectively. The Vi backbone content was determined using HPAEC-PAD (see *S.1.5*), and *O*-acetyl by the Hestrin assay. *C. freundii* Vi samples for analysis by NMR were freeze-dried.

### 2.3 Vi polysaccharide ELISAs

Vi ELISAs were performed in various formats: (1) co-coating with methylated human serum albumin (mHSA ELISA) [21], (2) pre-coating with Poly-L-Lysine (PLL ELISA) [21], and (3) by capture with an anti-Vi mAb (Capture ELISA). Details of the ELISA methods are included in Supplementary Sections 1.2–1.4.

### 2.4 Hestrin assay

A modified microplate version of the Hestrin method was used to determine the level of *O*-acetylation of Vi [24]. Fifty µL of Vi were added to wells of a microtiter plate (Nunc Maxisorp). Samples were diluted to 200 µg/mL Vi and a standard curve was made using acetylcholine chloride (Sigma, A6625) in 1 mM sodium acetate (Sigma, 71183) at concentrations of 0.055, 0.1375, 0.275, 0.550 and 1.375 µmol/mL acetylcholine chloride. Samples and standards were added in triplicate. A volume of 50 µL of 4 M HCl was added to the wells and mixed. Then 100 µL of alkaline hydroxylamine solution (made from equal volumes of 13.9% w/v hydroxylamine hydrochloride (Sigma, 255580) and 17.5% w/v NaOH, Fisher, S/4940/17) and was added and mixed. An additional 50 µL of 4 M HCl (VWR, 20255.290) was added and mixed. Finally, 50 µL 0.37 M FeCl_3_ (Sigma, F2877) was added and optical density readings taken at 540 nm using a Labsystems Multiskan plate reader. The concentration of *O*-acetylation in the sample was calculated directly from the standard curve given by linear regression of the plot of the absorbance versus micromole/mL acetylcholine and expressed as % mol *O*-acetyl/mol Vi repeating unit using the appropriate functional weight (for example, 216.2 g/mol for de-*O*-acetylated, 259.1 g/mol for *O*-acetylated) for the free acid form of Vi. Assay precision (12.0% CV) was determined from two different Vi samples run in triplicate in two separate assays [15].

### 2.5 NMR

Nuclear magnetic resonance (NMR) spectroscopy was performed with a Bruker 700 MHz spectrometer (Bruker, UK) fitted with a BBI RT probe. Before measurement, samples were exchanged/lyophilized three times, each time resuspending the polysaccharide in 600 µL D_2_O (Sigma, 151882). The *O*-acetylation level of control (untreated), 0.05 M and 1.5 M NH_4_OH-treated *C. freundii* Vi solutions were investigated by one-dimensional ^1^H and bi-dimensional ^1^H–^13^C Heteronuclear single quantum coherence (HSQC) spectroscopy analyses. For the control and in the weaker base-treated samples, signals in the one-dimensional ^1^H spectra are overlapping and too broad to be used for quantification, as previously described [25], [26]. ^1^H–^13^C HSQC was used for quantification, as both *N*- and *O*-acetyl signals are well-resolved in the two-dimensional plot. The area under the *N*- and *O*-acetyl cross-peaks (^1^H at 2.20 and 2.22 ppm, respectively) was used to determine the ratio of *O*- to *N*-acetylation, as it was not possible to correlate *O*- and *N*-acetyl groups to the anomeric ring signals (due to the low amount of Vi). One dimensional NMR measurements used a 1D-NOESY with pre-saturation (noesypr1d) and a mixing time of 10 ms. ^1^H–^13^C HSQC two-dimensional experiments were performed using the hsqccetgpsisp 2.2 pulse sequence, with a ^1^J(CH) coupling constant of 145 Hz. Processing was done using Topspin 4.0.1 (Bruker, UK).

### 2.6 Molecular modelling

All simulations were performed with the NAMD molecular dynamics program [27] version 2.12 with CUDA extensions [28]. Carbohydrates were modelled with the CHARMM36 additive force field for carbohydrates [29], [30], incorporating ad hoc extensions for the acetyl units. The TIP3P water model [31] was used to model aqueous solution.

We performed 550 ns molecular dynamics (MD) simulations of six repeating units (6RU) fragments of both a fully *O*-acetylated (Vi_6RU) and fully de-*O*-acetylated (Vi_deAc_6RU) Vi. The conformation of the α(1 → 4) linkage is described by the torsion angles φ = H1-C1-O4′-C4′ and ψ = C1-O1-O4′-H4′. Initial structures for both simulations were built with optimal torsional angle values determined from a φ, ψ potential of mean force calculation on a α(1 → 4) *N*-acetylgalacturonic disaccharide in vacuum. Model structures were generated with our CarbBuilder software [32], [33]. Simulations began with 10 000 steps of NAMD minimization in vacuum, followed by solvation in a 60 Å^3^ periodic water box (9260 water molecules) using the *solvate* routine in the Visual Molecular Dynamics (VMD) software [34]. Six randomly-distributed sodium ions were added to each system using the VMD *autoionize* feature in order to electrostatically neutralize the carboxyl groups. The MD simulations were preceded by a minimization-and-heating phase, comprising 5 K temperature reassignments from 10 K to 300 K, with 500 steps of minimization and 8000 steps of MD at each temperature. The Vi_6RU and Vi_deAc_6RU MD simulations were run for 550 ns.

Equations of motion were integrated using a Leap-Frog Verlet integrator with a step size of 1 fs and periodic boundary conditions. Simulations were performed under isothermal-isobaric (nPT) conditions at 300 K maintained using a Langevin piston barostat and the Nose-Hoover thermostat implemented in NAMD. Long-range electrostatic interactions were treated with particle mesh Ewald [35] summation using k = 0.20 Å^−1^ and PME grid dimensions equal to the system periodic cell dimensions. Non-bonded interactions were truncated with a switching function applied between 12.0 and 15.0 Å. The 1–4 interactions were not scaled.

### 2.7 Post-simulation analysis

Molecular structures were visualized with VMD [34]. Molecular conformations at intervals of 25 ps were extracted for analysis, discarding the first 100 ns of each simulation. End-to-end distances for the 6RU saccharide chains were defined from C-1 of residue 1 to C-4 of residue 6. For clustering analysis, the 6RU simulation conformations were aligned on the ring carbons of the middle 2RU and then clustered into conformational families using VMD’s internal *cluster* command, which applies the quality threshold algorithm [36]. The clustering metric comprised an RMSD fit of the non-hydrogen atoms for the middle 4RU of the 6RU strand.

Representative 20RU static models of both Vi and de-*O*-acetylated Vi were built using CarbBuilder to generate a random allocation of φ, ψ dihedral angle conformations for the α(1 → 4) glycosidic linkages, in accordance with the populations extracted from the MD simulations.

## 3 Results

### 3.1 Characterization of the Vi PS panel

**Fig. 2:**
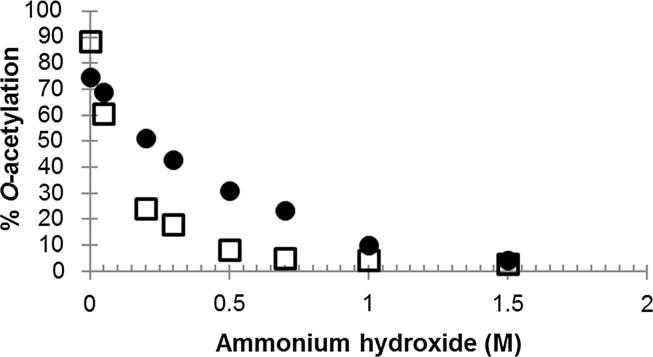
Effect of ammonium hydroxide treatment on the *O*-acetylation level of Vi polysaccharide standards from *C. fruendii* and *S.* Typhi. The points from control and de-*O*-acetylated Vi standard from *C. freundii* (●) and *S.* Typhi (□) are the means of values determined in two to four independent assays. Confidence intervals are included on untreated and 1.0 M base-treated. Base treatment was performed at 37 °C for 18 h, and *O*-acetylation was determined by the Hestrin method.

The *O*-acetylation levels determined from Hestrin assays (n = 5) confirmed that the *C. freundii* Vi standard was slightly less *O*-acetylated (75%) than the *S.* Typhi (88%, p = 0.05). Base treatment of the Vi with ammonium hydroxide resulted in a range of 4 to 75% *O*-acetylation for *C. freundii* Vi and 3 to 88% *O*-acetylation for *S.* Typhi Vi. In the 0.05 to 1 M ammonium hydroxide range, the *S.* Typhi Vi was significantly more susceptible to de-*O*-acetylation than *C. freundii* Vi (p < 0.001) (Fig. 2).

### 3.2 Effect of de-O-acetylation on Ab binding

**Fig. 3:**
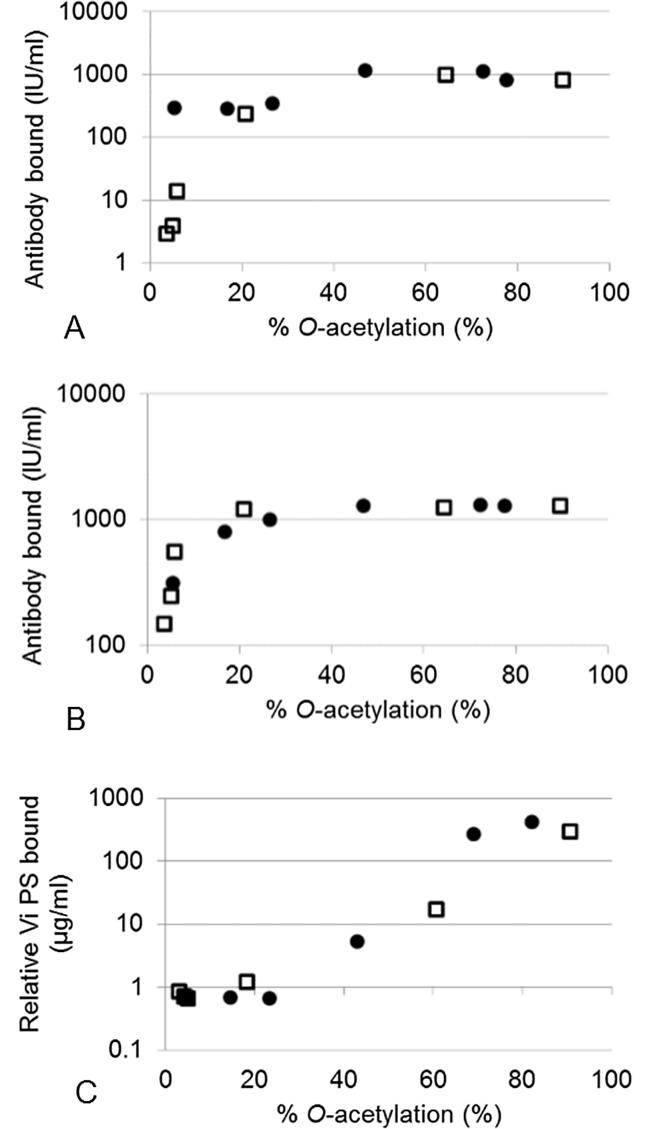
Effect of de-*O*-acetylation of Vi polysaccharide on the binding of anti-Vi IgG in mHSA and capture ELISAs. Post-immunization human 10/126 sera (A) and hyperimmune sheep sera (B) were used in mHSA ELISAs, and monoclonal mouse IgG was used in a capture ELISA (C). Data from control and de-*O*-acetylated Vi standard from *C. freundii* (●) and *S.* Typhi (□) are the mean of values determined in two independent assays (n = 4 data points).

Patterns of binding of the post-immunization human sera and sheep hyperimmune sera to the panel of Vi correlated with the level of *O*-acetylation and hence its source (see Supplementary for multi-dilution binding data). Human anti-Vi IgG bound equally to *C. freundii* Vi with 40 or 70% *O*-acetylation, and an equivalent amount of IgG bound to Vi with 30% *O*-acetylation or lower in the mHSA ELISA (Figs. 3A and S1). Human sera also bound in a titration-dependent manner to *S.* Typhi Vi at < 20% *O*-acetylation. Interestingly, Vi 64 to 72% *O*-acetylated was recognized slightly better than the ‘fully *O*-acetylated’ standards, a phenomenon also reported by Szu et al. [9].

This correlation between *O*-acetylation and IgG binding was also observed for sheep anti-Vi IgG (Figs. 3B and S1). As with human anti-Vi IgG, two or more phases of binding were observed: at > 20% *O*-acetylation there was equivalent binding to Vi, while at < 20% *O*-acetylation a relatively large decrease in binding was observed with decreasing *O*-acetylation.

While the *O*-acetyl is clearly a dominant epitope for both anti-Vi sera, as demonstrated by the loss of most of the IgG binding upon de-*O*-acetylation, binding of anti-Vi IgG also occurred in low- or non-*O*-acetylated polysaccharide. This is suggestive of the presence of epitope(s) that are independent of the *O*-acetyl group.

A mouse anti-Vi mAb was used to evaluate the presence of *O*-acetyl epitopes on treated and untreated Vi. Only Vi captured by this Mab could be detected by the secondary anti-Vi serum, and the correlation between *O*-acetylation and binding (r ≥ 0.8) confirms that the mAb only binds to the *O*-acetyl group of Vi. In contrast to results of indirect ELISAs with polyclonal anti-Vi sera, the capture ELISA showed that Vi with *O*-acetylation levels below 65–70% had a significant decrease in bound IgG and Vi with *O*-acetylation levels below 25% was not detected at all (Figs. 3C and S2). The high sensitivity of this mAb for *O*-acetylation of Vi makes it a perfect tool to determine the *O*-acetylation level of Vi and shows that the capture ELISA could be an alternative to the Hestrin test.

**Fig. 4:**
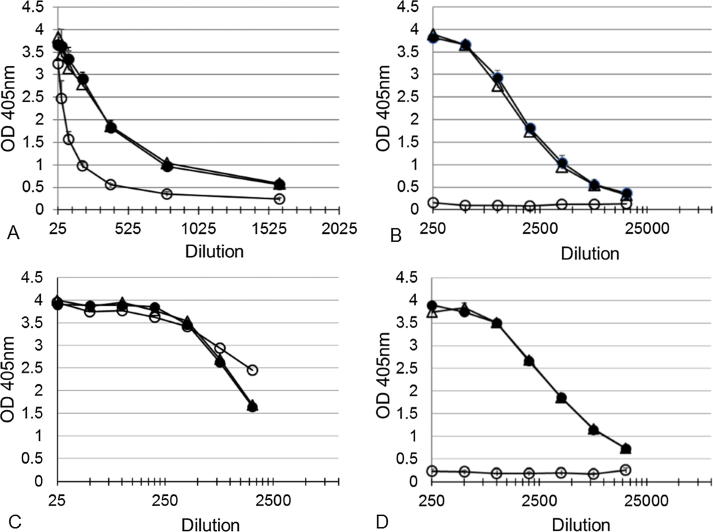
Effect of de-*O*-acetylation of Vi polysaccharide from *C. freundii* (A and B) and *S.* Typhi (C and D) on binding to human anti-Vi IgG 16/138 (A and C) and mouse monoclonal anti-Vi IgG (B, D) by PLL ELISA. Untreated (●), 0.05 M (△) and 1.5 M (○) ammonium hydroxide-treated Vi PS was co-coated with PLL to the plates. The results are the average of two assays and error bars represent standard deviations.

These observations were confirmed by a third ELISA, the PLL ELISA. As with the mHSA ELISA, human anti-Vi IgG binding (using the WHO 1st IS) was not completely abolished for the fully de-*O*-acetylated Vi (Fig. 4).

### 3.3 Further characterization of *C. Freundii* and *S. Typhi* Vi PS standards

**Fig. 5:**
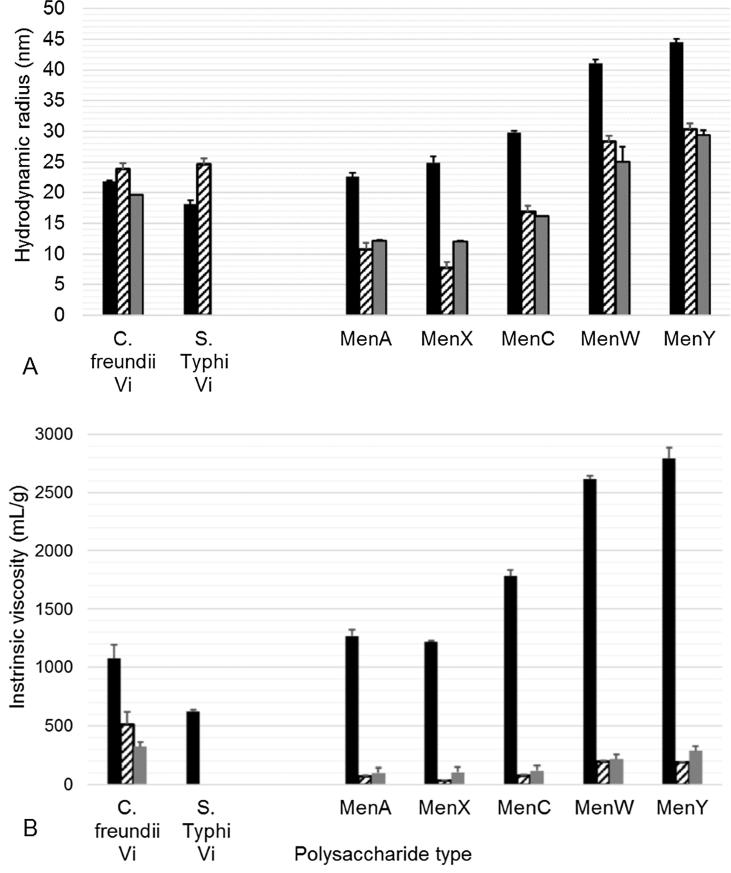
Hydrodynamic behavior of Vi and meningococcal polysaccharide standards by SEC/MALS/Viscometry. The hydrodynamic volume (A) and intrinsic viscosity (B) of the PSs were determined during their chromatography on a TSKgel G5000PW_XL_ column in water (black bar), 20 mM Hepes, pH 7.4 (striped bar) or PBS, pH 7.4 (gray bar). Fifty µg of each PS standard were loaded in each case. Standard deviations of three replicate injections are shown by error bars.

Some differences were seen between the binding of human sera to de-*O*-acetylated *C. freundii* and *S.* Typhi Vi, which may be related to differing viscosities and/or self-association. We further characterized their solution behavior with SEC-MALS-RI-Viscometry, using meningococcal polysaccharides as comparators, which are known to be relatively viscous in high concentrations (method details and chromatograms are given in the Supplementary). The elution profiles of the PSs were eluent-dependent (Fig. S3). In water, the polysaccharides of both Vi and Meningococcal PS had higher hydrodynamic radii and viscosity (i.e. were more swollen), than in 20 mM Hepes or phosphate-buffered saline (PBS) at neutral pH (Fig. 5A and B). In buffer, cations may complex with the anionic phosphorus (MenA and X PSs) or carboxyl groups (Vi, and MenC, W and Y PSs) to form a more compact structure [37]. The *S.* Typhi Vi did not elute well with a PBS eluent and we found poor reproducibility of *S.* Typhi elution in Hepes buffer. Higher hydrodynamic radii and viscosity values were determined for the *C. freundii* Vi than for the *S.* Typhi standard in water, which may explain their differences in base susceptibility, although this was not observed in buffers with higher ionic strength.

We found no significant difference in the weight average molecular mass (Mw) of the two Vi standards, although these were eluent dependent. The Mw values for the *C. freundii* Vi were 73,000 g/mol (water), 203,000 g/mol (Hepes) and 194,000 g/mol (PBS). De-*O*-acetylated *C. freundii* Vi had the same Mw as untreated PS, and slightly lower viscosity (data not shown). *C. freundii* Vi had a molecular mass of 165 kDa in phosphate buffer containing acetonitrile using dextran standards [38]. The *S.* Typhi Vi standard had Mw values of 70,000 and 224,000 g/mol in water and Hepes, respectively. In PBS, Mw for the meningococcal PS ISs were: 141,000 g/mol, MenA; 135,000 g/mol, MenX; 271,000 g/mol, MenC; 604,000 g/mol, MenW; and 816,000 g/mol, MenY. Higher intrinsic viscosities (and mass values) for MenY and MenW than the other serogroups have been reported by Abdelhameed et al. [39]. The intrinsic viscosities of the Vi were higher than the meningococcal PS, except in water.

With a PL Aquagel-OH 60 column, we obtained poorer recovery of the Vi and patterns of elution indicative of non-ideal interaction with the beads. Giannelli et al. found acetonitrile improved recovery [17], but the inclusion of this was problematic with SEC-MALS, with a dampening of MALS signals.

### 3.4 Exploration of N- and O-acetylation by HSQC NMR

**Fig. 6:**
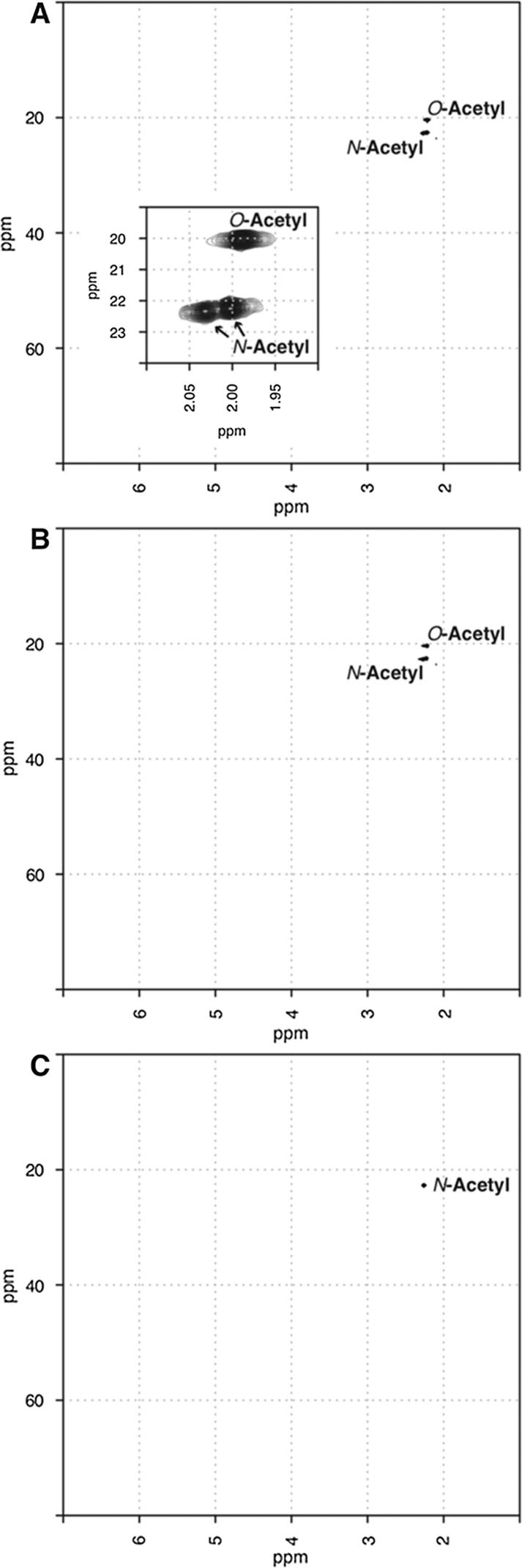
*O*- and *N*-acetylation signals detected by ^1^H–^13^C HSQC NMR analysis of Vi polysaccharide solutions. Bi-dimensional NMR spectra of (A) untreated, (B) 0.05 M NH_4_OH-treated, and (C) 1.5 M NH_4_OH-treated *C. freundii* Vi solutions. The x- and y-axis contain the NMR resonances arising from ^1^H and ^13^C, respectively. The insert in panel A has an expanded region of the acetyl crosspeaks.

NH_4_OH-treated *C. freundii* Vi solutions were analyzed by NMR to check the level of residual *O*-acetylation. The *O*-acetylation level of control (untreated), 0.05 M and 1.5 M NH_4_OH-treated Vi solutions were investigated by one-dimensional ^1^H and bi-dimensional ^1^H–^13^C HSQC NMR analyses. In the control and in the weaker base-treated samples, signals in the one-dimensional ^1^H spectra are overlapping and too broad to be used for quantification, as previously described [25], [26]. ^1^H–^13^C HSQC was used for quantification, as both *N*- and *O*-acetyl signals are strong and well-resolved in the bi-dimensional plot (Fig. 6A and B). The area under the *N*- and *O*-acetyl cross-peaks (^1^H at 2.20 and 2.22 ppm, respectively) was used to determine the ratio of *O*- to *N*-acetylation, as it was not possible to correlate *O*- and *N*-acetyl groups to the anomeric ring signals (due to the low amount of Vi). In the bi-dimensional spectrum of the untreated Vi, evidence of 100% *N*-acetylation was found, as 2 crosspeaks for the *N*-acetyl group and only 1 crosspeak for the *O*-acetyl group were detected. The detection of these crosspeaks is a clear indication of total *N*-acetylation and partial *O*-acetylation in the untreated native Vi.

In the case of the strongest base-treated sample (1.5 M NH_4_OH), signals of Vi in the one-dimensional ^1^H spectra are better resolved and sharp enough to be used for quantification (data not shown). In addition, no *O*-acetyl groups were detected by ^1^H–^13^C HSQC (Fig. 5C). So, the H-1 signal was used to determine the percentage of *N*-acetylation after basic treatment. Integration of the H-1 and *N*-acetyl peaks showed no loss in *N*-acetylation as expected from the treatment employed to effect de-*O*-acetylation.

In the 1.5 M NH_4_OH-treated Vi, as previously reported, the signals from the GalNAcA sugar ring were more detectable after basic treatment in the ^1^H–^13^C HSQC plot. By NMR, 75.1% and 55.7% *O*-acetylation were determined for the untreated and mild-base treated samples, respectively, compared with 72 and 66% for the same samples using the micro-Hestrin method. In the strongest base-treated sample, *O*-acetylation could not be measured by NMR, while 7.6% was determined by the Hestrin assay. We cannot rule out without further analysis that polysaccharide backbone conformation modifications occurred as a result of de-*O*-acetylation.

### 3.5 MD simulations of the conformation and dynamics of Vi PS

**Fig. 7:**
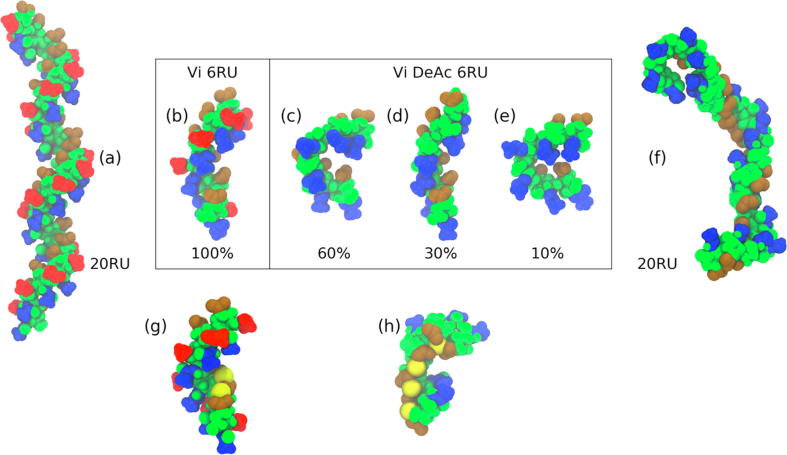
Conformations of the *O*-acetylated (left) and de-*O*-acetylated (right) Vi polysaccharide. The box shows representative structures for the dominant conformational families for the MD simulations 6RU: the acetylated strand was in a single helical conformation (b) for the entire simulation, while the de-*O*-acetylated strand alternated between the bent conformations shown in (c) and (d) and a helical conformation (d). The 6RU Vi PS helix can intercalate 1 to 2 sodium ions (g), whereas the more flexible de-*O*-acetylated strand intercalated up to 4 ions (h). The corresponding 20RU static models are shown for (a) Vi PS and (f) de-*O*-acetylated Vi. Atoms from the constituent groups are highlighted as follows: *N*-acetyl, ; *O*-acetyl, ; carboxyl, , with sodium ions,  and the remaining atoms colored .

MD simulations in aqueous solution indicate that 6RU of the 100% *O*-acetylated Vi presents a single helical conformational epitope (Fig. 7b). This helical conformation is present for 100% of the 550 ns simulation and is markedly more constrained than reported for a short 5 ns simulation of a fully acetylated 6 RU strand with the general AMBER force field [40]. The *O*-acetyl groups (highlighted in red in Fig. 7) are solvent exposed on the helical surface and thus are likely to be a primary site of Ab binding. In contrast, the *N*-acetyls and the negatively charged carboxyl groups are relatively hidden from the solvent on opposite edges of the helical cavity.

In contrast, 6RU of completely de-*O*-acetylated Vi PS is more flexible, showing a wide range of conformational families, with “bent” conformations (Fig. 7c and e) dominating over the helical conformation (Fig. 7d). These *N*-acetyl and carboxyl groups are more exposed in the dominant bent conformation (Fig. 7c) than in the 100% *O*-acetylated polysaccharide.

The carboxyl groups have the potential to intercalate sodium ions in Vi. The fully *O*-acetylated 6RU strand transiently traps one to two sodium ions during the course of the simulation (Fig. 7g). This interaction of the carboxyl groups with free sodium ions is considerably increased with de-*O*-acetylation: the 6RU of completely de-*O*-acetylated Vi intercalated up to four sodium ions during the simulation (Fig. 7h). Thus, increasing de-*O*-acetylation may increase the hydrodynamic radius of the Vi PS, because of the close association of sodium (or other) cations. The phenomenon may also be enhanced for all Vi with increasing ionic strength of the solution - with higher ionic strength, the helical channel in fully *O*-acetylated Vi may be fully occupied with ions.

The 20RU static models of both acetylated Vi (Fig. 7a) and de-*O*-acetylated Vi (Fig. 7f) provide a prediction and visualization, on the basis of our 6RU MD simulations, of the conformation of the corresponding polysaccharide. For this longer strand, Vi forms a regular helix, whereas the de-*O*-acetylated Vi has helical stretches interspersed with more disorganized regions.

**Fig. 8:**
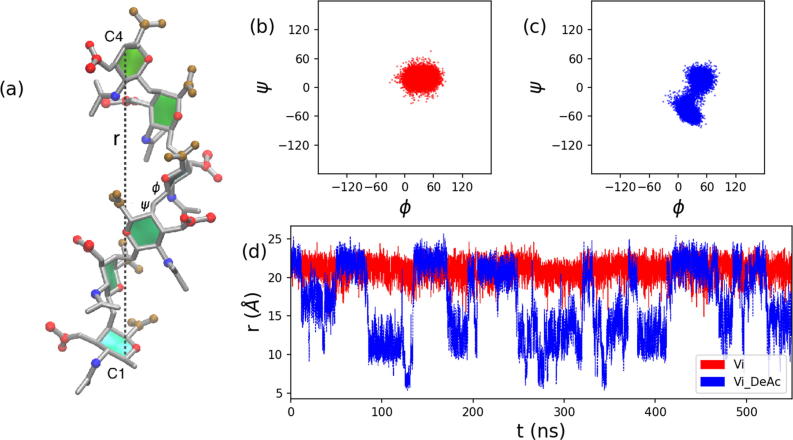
Comparison of the simulation dynamics in *O*-acetylated (red) and de-*O-*acetylated (blue) Vi polysaccharide. (a) An example conformation of *O*-acetylated Vi with the C1-C4 end-to-end distance, *r,* and the φ and ψ dihedral angles labeled. Atoms are highlighted as follows: *N*-acetyl, ; O-acetyl, ; carboxyl, . Simulation time series of the φ and ψ dihedral angles show differing conformations for the glycosidic linkage in (b) *O*-acetylated Vi and (c) de-*O*-acetylated Vi, with the de-*O*-acetylated strand rotating more freely (d). The same is true for the chain extension: the *r* end-to-end distance time series is relatively constant for *O*-acetylated Vi () and much more variable in de-*O*-acetylated Vi ().

Acetylation changes not only the conformation, but also the flexibility of the Vi polymer. The conformation of the α(1 → 4) glycosidic linkage in polysaccharides is commonly described by two dihedral angles: φ and ψ (shown on a representative disaccharide linkage in Fig. 8a). Comparison of the φ, ψ scatter plots for the 6RU simulations for Vi (Fig. 8b) and de-*O*-acetylated Vi (Fig. 8c) reveals that the *O*-acetyl groups function to lock the glycosidic linkage into a single conformation, producing a helix for 6RU Vi. This helix has little mobility: it is much closer to a rigid rod than a flexible coil. The *O*-acetyl substitutions block φ, ψ rotations through steric collisions with the *N*-acetyl group on the neighboring residue. This rigidity provides a clear conformational rationale for the known viscosity of *O*-acetylated Vi [11], [14], [41], [42]. With *O-*acetyls fully removed, a larger range of linkage rotations becomes possible and the saccharide consequently becomes much more flexible and mobile. This increased mobility and comparative dynamic motion of the de-*O*-acetylated chain is clearly seen in a comparison of the time series for the end-to-end distance throughout the simulation (Fig. 7D): Vi maintains a consistent extended helix (red line), whereas de-*O*-acetylated Vi (blue line) alternates regularly between extended and more compressed conformations. The flexibility of de-*O*-acetylated Vi compared to fully *O*-acetylated Vi accounts for the sharper lines observed in the NMR spectra after de-*O*-acetylation.

## 4 Discussion

While two International Standards established for the quantitation of Vi polysaccharide in conjugated and unconjugated vaccines have been shown to be of vaccine quality and have similar stability, we show here that there are slight differences in the chemical and physical behavior of the Vi standards derived from *C. freundii* and *S. Typhi*. In particular, the Vi of *C. freundii* is relatively more resistant to base-catalyzed de-*O*-acetylation and has a larger hydrodynamic radius and higher viscosity in water than the *S.* Typhi Vi. MD simulations indicate that de-*O*-acetylation has a marked effect on the conformation and dynamic behavior of the Vi, changing the capsular polysaccharide from a rigid helix into a more flexible coil. Extrapolation of these results suggests that the more highly *O*-acetylated the chain, the more helical and defined the conformation and hence the more viscous the solution.

In column buffers containing sodium as a counterion, there is evidence of a more compact polysaccharide helix, and the hydrodynamic volumes are similar. As a reference standard in quantitative assays such as HPAEC-PAD and rocket immuno-electrophoresis, the use of the *C. freundii* quantitative standard gave slightly lower measured Vi saccharide contents for *S.* Typhi Vi unconjugated and conjugated vaccines than did the homologous Vi [15], [16], further demonstrating a slight species-specificity in the behavior and supra-structure of the Vi in solution. Although both standards were purified to contain sodium as the main counterion, differences in their extraction cannot be ruled out, and high-affinity cations can be difficult to completely remove [5]. Our MD simulations indicated strong interaction of the Vi with sodium ions, which increases with de-*O*-acetylation; cations are tightly held and generate a larger hydrodynamic radius for the polysaccharide. When *O*-acetylation levels were accounted for, the anti-Vi IgG binding patterns in both standards were similar.

The role of *O*-acetylation and its criticality for participating in the protective epitopes of bacterial capsular PS differ for each; Berti et al. hypothesized that *O*-acetyls positioned (or oriented) within the saccharide main ring, as is the case for Vi, may be more critical for immunoprotection [43]. We demonstrated binding of the anti-Vi IgG in polyclonal human and sheep immune sera to de-*O*-acetylated Vi of both species. However minimal compared to *O*-acetyl-dependent binding, this is a demonstration of alternative epitopes on Vi. Binding of human anti-Vi IgG to de-*O*-acetylated Vi has not previously been described. A mAb with specificity to a non-*O*-acetyl epitope, purported to bind to the carboxyl group [44] and additional epitopes on Vi involving carboxyl and/or *N*-acetyl groups, have been reported for de-*O*-acetylated Vi [9], [12].

Our molecular simulations reveal that the carboxyl group is not likely to form a dominant epitope because of its low solvent exposure in the Vi polysaccharide helix, even in the non-*O*-acetylated model. The *O*-acetyl groups are highly solvent exposed on the helical surface and therefore a prime site for binding, with the carboxyl and *N*-acetyl groups comparatively hidden in the helical cavity; only with sufficient de-*O*-acetylation (50 to 80%), are other epitopes revealed. The experimental evidence of slightly higher antibody binding to the partially *O*-acetylated Vi than fully *O*-acetylated form can be explained by a slight increase in backbone flexibility facilitating antibody binding. From a vaccine point-of-view, it is interesting that partially *O*-acetylated polysaccharide may potentially be advantageous in revealing additional epitope(s) and possibly reduced viscosity and hydrophobicity. Cation-dependent stabilization of the partially *O*-acetylated Vi helix leads to different conformers in our simulations, which could explain the polyclonality of antisera, as well as the potential for maturation of the immune response (for example, additional epitopes could be exposed on the polysaccharide backbone following vaccine administration and loss of *O*-acetyl groups *in situ*).

It is almost forty years since Vi polysaccharide-based vaccines were introduced, and improvements in our understanding of the nature of this virulence antigen are still forthcoming. The high viscosity and rigidity of the Vi, due in large part to *O*-acetylation, has bearing on vaccine manufacture, purification, and analysis. Vi PS-vaccine shortages between 2012 and 2017 signaled manufacturing challenges; production of the conjugate vaccines also depends upon the purification of Vi, though conjugation is likely to reduce viscosity [39]. Laboratories utilizing Vi standards and antisera should be aware of the impact of cations in buffers used for Vi. Immunogenicity studies should include a panel of partially-to-fully *O*-acetylated Vi preparations to explore more precisely the extent of *O*-acetylation required for optimal and boost-able responses.

## Contributors

All authors conceptualized aspects of the study, and the research was performed by PH (Hestrin), KH and EB (ELISA), KL, GDE, BB (MALS), GDE (NMR) and MK (Molecular Modelling and analysis). BB, SR, GDE, and MK prepared the manuscript, with participation by the other co-authors. All have approved the final article.

## Funding

Funding for assay development and optimization was given by the Coalition Against Typhoid of the Sabin Vaccine Institute to SR which funded PH, and by the World Health Organization to FG. The South African National Research Foundation (Grant no: 103805) provided funding for the molecular modelling component of this work. KH and EB were supported by the Bill & Melinda Gates Foundation (OPP 1145952).

## Declaration of Competing Interest

Authors from NIBSC have none to declare.

## References

[1] Felix A., Pitt R.M. (1934). A new antigen of B. typhosus. Its relation to virulence and to active and passive immunisation. Lancet.

[2] Robbins J.D., Robbins J.B. (1984). Re-examination of the protective role of the capsular polysaccharide (Vi antigen) of *Salmonella* typhi. J Infect Dis.

[3] Clark W.R., McLaughlin J., Webster M.E. (1958). An aminohexuronic acid as the principal hydrolytic component of the Vi antigen. J Biol Chem.

[4] Heyns K., Kiessling G. (1967). Strukturaufklärung des Vi-antigens aus *Citrobacter freundii* (*E. coli*) 5396/38. Carb Res.

[5] Bystricky S., Szu S.C. (1994). *O*-acetylation affects the binding properties of the carboxyl group on the Vi bacterial polysaccharide. Biophys Chem.

[6] Szu S.C. (2013). Development of Vi conjugate - a new generation of typhoid vaccine. Exp Rev Vacc.

[7] WHO. Requirements for Vi polysaccharide typhoid vaccine. In: 43rd meeting of the WHO Expert Committee on Biological Standardization. WHO Tech Rep Series 1994; 840:14-33 (Annex 1). http://apps.who.int/iris/bitstream/handle/10665/42058/WHO_TRS_872.pdf?sequence=1.

[8] WHO. Guidelines on the quality, safety and efficacy of typhoid conjugate vaccines. In: 64th meeting of the WHO Expert Committee on Biological Standardization. WHO Tech Rep Series 2014; 987:101-173 (Annex 3). http://www.who.int/biologicals/areas/vaccines/TYPHOID_BS2215_doc_v1.14_WEB_VERSION.pdf Accessed 08 January 2014.

[9] Szu S.C., Li X.R., Stone A.L., Robbins J.B. (1991). Relation between structure and immunologic properties of the Vi capsular polysaccharide. Infect Immun.

[10] Rijpkema S., Durrani Z., Lemercinier X., Jones C. (2004). Detection of *O*-acetylated Vi polysaccharide of *Salmonella enterica* subspecies typhi by enzyme immunoassay. Biologicals.

[11] Jarvis F.G., Mesenko M.T., Martin D.G., Perrine T.D. (1967). Physicochemical properties of the Vi antigen before and after mild alkaline hydrolysis. J Bacteriol.

[12] Szewczyk B., Taylor A. (1980). Immunochemical properties of Vi antigen from Salmonella typhi Ty2: presence of two antigenic determinants. Infect Immun.

[13] Landy M., Gaines S., Seal J.R., Whiteside S.E. (1954). Antibody responses of man to three types of antityphoid immunizing agents: Heat-phenol fluid vaccine, acetone-dehydrated vaccine, and isolated Vi and O antigens. Am J Public Health Nations Health.

[14] Szu S.C., Stone A.L., Robbins J.D., Schneerson R., Robbins J.B. (1987). Vi capsular polysaccharide-protein conjugates for prevention of typhoid fever. Preparation, characterization, and immunogenicity in laboratory animals. J Exp Med.

[15] WHO. Evaluation of candidate International Standards for Vi polysaccharide from Citrobacter freundii and *Salmonella enterica* subspecies *enterica* serovar Typhi. In: Expert Committee On Biological Standardization, Geneva, 17 to 20 October 2017 (WHO/BS/2017.2310). http://www.who.int/biologicals/expert_committee/BS2310_Vi_PS_Report_for_WHO_Final.pdf?ua=1 Accessed 22 March, 2017. 10.1016/j.biologicals.2018.11.004 30502020

[16] Gao F., Swann C., Rigsby P., Rijpkema S., Lockyer K., Logan A. (2019). Evaluation of two WHO first international standards for Vi polysaccharide from *Citrobacter freundii* and *Salmonella enterica* subspecies *enterica* serovar Typhi. Biologicals.

[17] Giannelli C., Cappelletti E., Di Benedetto R., Pippi F., Arcuri M., Di Cioccio V. (2017). Determination of free polysaccharide in Vi glycoconjugate vaccine against typhoid fever. J Pharm Biomed Anal.

[18] Hlozek J., Kuttel M.M., Ravenscroft N. (2018). Conformations of *Neisseria meningitidis* serogroup A and X polysaccharides: the effects of chain length and O-acetylation. Carb Res.

[19] Vipond C., Mulloy B., Rigsby P., Burkin K., Bolgiano B. (2012). MenC IS working group. evaluation of a candidate international standard for meningococcal group C polysaccharide. Biologicals.

[20] Vipond C., Swann C.J., Dougall T.W., Rigsby P., Gao F., Beresford N.J., Bolgiano B. (2017). The MenA/MenX IS working group. evaluation of candidate international standards for meningococcal serogroups A and X polysaccharide. Biologicals.

[21] Rijpkema S., Hockley J., Logan A., Rigsby P., Atkinson E., Jin C., Pasetti M.F., Pollard A.J. (2018). The anti-Vi IgG working group. Establishment of the first international standard for human anti-typhoid capsular Vi polysaccharide IgG. Biologicals.

[22] Jin C., Gibani M.M., Moore M., Juel H.B., Jones E., Meiring J. (2017). Efficacy and immunogenicity of a Vi-tetanus toxoid conjugate vaccine in the prevention of typhoid fever using a controlled human infection model of *Salmonella* Typhi: a randomised controlled, phase 2b trial. Lancet.

[23] Lin F.Y., Ho V.A., Khiem H.B., Trach D.D., Bay P.V., Thanh T.C. (2001). The efficacy of a *Salmonella* typhi Vi conjugate vaccine in two-to-five- year-old Children. N Engl J Med.

[24] Hestrin S. (1949). The reaction of acetylcholine and other carboxylic acid derivatives with hydroxylamine, and its analytical application. J Biol Chem.

[25] Daniels D.M., Schneerson R., Egan W.H., Szu S.C., Robbins J.B. (1989). Characterization of the *Salmonella paratyphi* C Vi polysaccharide. Infect Immun.

[26] Lemercinier X., Martinez C.I., Jones C. (2000). Use and validation of an NMR test for the identity and *O*-acetyl content of the *Salmonella* typhi Vi capsular polysaccharide vaccine. Biologicals.

[27] Phillips J.C., Braun R., Wang W., Gumbart J., Tajkhorshid E., Villa E. (2005). Scalable molecular dynamics with NAMD. J Comput Chem.

[28] Stone J.E., Phillips J.C., Freddolino P.L., Hardy D.J., Trabuco L.G., Schulten K. (2007). Accelerating molecular modeling applications with graphics processors. J Comput Chem.

[29] Guvench O., Greene S.N., Kamath G., Brady J.W., Venable R.M., Pastor R.W. (2008). Additive empirical force field for hexopyranose monosaccharides. J Comput Chem.

[30] Guvench O., Hatcher E., Venable R.M., Pastor R.W., MacKerell A.D. (2009). CHARMM additive all-atom force field for glycosidic linkages between hexopyranoses. J. Chem. Theory Comput..

[31] Jorgensen W.L., Chandrasekhar J., Madura J.D., Impey R.W., Klein M.L. (1983). Comparison of simple potential functions for simulating liquid water. J Chem Phys.

[32] Kuttel M.M., Ståhle J., Widmalm G. (2016). CarbBuilder: Software for building molecular models of complex oligo- and polysaccharide structures. J Comput Chem.

[33] Kuttel M., Mao Y., Widmalm G., Lundborg M. (2011). CarbBuilder: an adjustable tool for building 3D molecular structures of carbohydrates for molecular simulation. Proceedings of 7^th^ IEEE International Conference on e-Science Stockholm, Sweden.

[34] Humphrey W., Dalke A., Schulten K. (1996). VMD: visual molecular dynamics. J Mol Graph.

[35] Darden D., York D., Pedersen L. (1993). Particle mesh Ewald: an *N*·log(*N*) method for Ewald sums in large systems. J Chem Phys.

[36] Heyer L.J., Kruglyak S., Yooseph S. (1999). Exploring expression data: Identification and analysis of coexpressed genes. Genome Res.

[37] Hadidi M., Buckley J.J., Zydney A.L. (2016). Effects of solution conditions on characteristics and size exclusion chromatography of pneumococcal polysaccharides and conjugate vaccines. Carbohydr Polym.

[38] Arcuri M., Di Benedetto R., Cunningham A.F., Saul A., MacLennan C.A., Micoli F. (2017). The influence of conjugation variables on the design and immunogenicity of a glycoconjugate vaccine against *Salmonella* Typhi. PLoS ONE.

[39] Abdelhameed A.S., Adams G.G., Morris G.A., Almutairi F., Adams G.G., Duvivier P. (2016). Solution conformation and flexibility of capsular polysaccharides from *Neisseria meningitidis* and glycoconjugates with the tetanus toxoid protein. Sci Rep.

[40] Legnani L, Compostella F, Grazioso G, Albini FM, Toma, L. Molecular dynamics simulations of the Salmonella typhi Vi antigenic polysaccharide and effects of the introduction of a zwitterionic matrix. Organic & Biomolecular Chem 9:pp. 5554–5559. 10.1039/c1ob05617d 21701726

[41] Landy M., Johnson A.G., Webster M.E. (1961). Studies on Vi antigen: the role of acetyl in antigenic activity. Am J Hyg.

[42] Stone A.L., Szu S.C. (1988). Application of optical properties of the Vi capsular polysaccharide for quantitation of the Vi antigen in vaccines for typhoid fever. J Clin Microbiol.

[43] Berti F., De Ricco R., Rappuoli R. (2018). Role of *O*-acetylation in the immunogenicity of bacterial polysaccharide vaccines. Molecules.

[44] Qadri A., Ghosh S., Talwar G.P. (1990). Monoclonal antibodies against two discrete determinants on Vi capsular polysaccharide. J Immunoassay.

