# Supplementary material for: O-acetylation of typhoid capsular polysaccharide confers polysaccharide rigidity and immunodominance by masking additional epitopes
Source: Vaccine. 2019 Jun 27;37(29):3866–75. doi: 10.1016/j.vaccine.2019.05.050 (PMC6997886; doi:10.1016/j.vaccine.2019.05.050)
Supplement: Supplementary data 1 [file mmc1.docx]

**Supplementary Material**

*Hitri et al., O-acetylation of typhoid capsular polysaccharide confers polysaccharide rigidity and immunodominance by masking additional epitopes*

**S.1.0. Methods**

*S.1.1. Antisera and antibodies*

| **Antibody** | **Species** | **Source** | **Catalog Number** | **Working Dilution** | **ELISA^a^** |
| --- | --- | --- | --- | --- | --- |
| WHO 1^st^ IS (Human Anti-Vi polyclonal serum)^b^ | Human | NIBSC | 10/126 | 1:50 | mHSA |
| WHO 1^st^ IS (Human Anti-Vi polyclonal serum)^c^ | Human | NIBSC | 16/138 | 1:25 | PLL |
| Anti-Vi polyclonal serum^d^ | Sheep | NIBSC | 07/160 | 1:500 | mHSA, Capture |
| Monoclonal Anti-Vi | Mouse | Oxford Biosystems | 15514 | 1:2000 | Capture, PLL |
| Anti-human IgG Fc-AP | Mouse | Abcam | Ab99764 | 1:5000 | PLL |
| Anti-mouse IgG Fc-AP | Goat | Abcam | Ab97262 | 1:5000 | PLL |
| Horse anti-mouse IgG | Horse | Pierce | 31181 | 1:1000 | Capture |
| Anti-sheep IgG-HRP | Donkey | Sigma | A3415 | 1:10000 | Capture |
| Anti-human IgG-HRP | Goat | Sigma | A8667 | 1:10000 | mHSA |
| Anti-mouse IgG-HRP | Rabbit | Sigma | A9044 | 1:10000 | mHSA |

^a^ mHSA, Capture and PLL ELISAs are described in sections 2.2, 2.3, and 2.4, respectively.

^b^ This human antiserum standard was prepared from adults who received two doses of Vi-TT vaccine. It has a unitage of 54 IU/ampoule, relative to the WHO 1^st^ IS
 10/16/138.

^c^ This human antiserum standard (WHO 1^st^ IS) was prepared from adults who received one dose of either Vi-TT or Vi PS vaccine. Sera was collected 4–6 weeks post-
 vaccination. It was assigned a unitage of 100 IU/ampoule [1].

^d^ The sheep antiserum was collected after four boosters of one single human dose of Vi conjugated to recombinant *Pseudomonas aeruginosa* exoprotein A (Vi-rEPA) in
 Freund’s adjuvant [2]. It has a potency of 1300 IU/mL relative to human antisera 16/138.

n.g. not given; AP, Alkaline Phosphatase-conjugated; HRP-Horse Radish Peroxidase-conjugated.

*S.1.2 Vi mHSA ELISA*

This indirect ELISA uses methylated human serum albumin (mHSA, NIBSC 12/176) as a co-coating agent for Vi and is a modification of the method developed by Arakere and Frasch [1,3]. In brief, wells of a micro-titre plate (Nunc Maxisorp) were coated with 100 µL containing a mixture of 5 µg/mL mHSA and 5 g/mL of native or base-treated Vi preparations from either *C. freundii* or *S. Typhi*. Anti-Vi sera from human or sheep were used in starting dilutions of 1:50 and 1:500, respectively. Bound IgG was detected by incubation with peroxidase-conjugated goat anti-human IgG (Sigma A8667) and peroxidase-conjugated donkey anti-sheep IgG (Sigma A3415) respectively. One hundred µL of TMBlue substrate (Leinco) was added to each well and the color reaction was stopped by adding 25 µL H_2_SO_4_. The plate was read at OD 450 nm. Duplicates were run on the same plate, and the average value and standard deviation taken from two independent assays (n=4) is presented in the Supplementary. CombiStats software (EDQM), was used to log transform and analyze assay responses as relative Vi PS bound by IgG.

*S.1.3 Vi Capture ELISA*

Wells of a micro-titer plate (Nunc Maxisorp) were coated with 100 µL horse anti-mouse IgG (Pierce) diluted 1:1000 in carbonate coating buffer (pH 9.6) and incubated overnight at 4 ^°^C. Plates were washed 3 times by hand with Washing Buffer (PBS: 10.1 mM sodium phosphate, 1.84 mM potassium phosphate, 171 mM NaCl, 3.4 mM KCl, pH 7.3-7.5, with 0.1% v/v Brij-35). 100 µL of monoclonal IgG anti-Vi PS diluted 1:2000 in Assay Diluent (AD: Washing Buffer and 1% w/v bovine serum albumin, BSA) was added to each well. Following incubation at room temperature on a shaker (550 rpm) for 2 hours, plates were washed, 250 µL blocking buffer (1% BSA in PBS was added to each well, plates were incubated at room temperature for 1 hour and washed by hand. Untreated and base-treated Vi preparations were diluted 1:100 to 1:500 in AD and 200 µL was added to the first wells in duplicate and 100 µL AD was added to adjacent wells. Doubling dilutions were prepared across the plate, leaving one column as blank. Plates were incubated at room temperature for 1 hour. Plates were washed and 100 µL of sheep anti-Vi serum diluted 1:500 in AD was added to each well. Plates were incubated at room temperature for 2 hours, washed and 100 µL of peroxidase-conjugated donkey anti-sheep IgG diluted 1:10000 in AD was added to each well. Plates were then incubated at room temperature for 1 hour and washed as described. Finally, 100 µL TMBlue substrate was added to each well and color was developed in the dark for 10 min. The reaction was stopped by adding 25 µL of H_2_SO_4_ to each well. The plate was read at 450 nm. Two replicates were run on the sample plate, and the average and standard deviation of two assays (n=4) was determined. Multi-dilution binding curves were evaluated by CombiStats [EDQM].

*S.1.4 Poly-L-Lysine ELISA*

An indirect ELISA was performed which utilizes Poly-L-Lysine hydrobromide (PLL; Sigma P1399) as a pre-coating agent for Vi from *C. freundii or S.* Typhi (untreated or de-*O*-acetylated). Wells were pre-coated with 100 µL of 10 μg/mL PLL for 2 hours at room temperature and washed 3 times with 0.9% w/v NaCl and 0.1% v/v Brij-35 detergent 0.3% w/v NaN_3_ in water (NaCl-Brij-NaN_3_), using a validated automatic plate washer. Plates were coated with 5 μg/mL Vi PS (untreated or de-*O*-acetylated) at 37 °C overnight and then washed 3 times with NaCl-Brij-NaN3. Non-specific binding sites were blocked with 200 μL 1% w/v BSA in PBS ‘A’ blocking buffer for 1 hour at 37 °C and washed 3 times with NaCl-Brij-NaN_3_ buffer. Working dilutions of human antisera or mouse monoclonal antibody were prepared in 1% BSA-0.1% Brij in PBS dilution buffer at a dilution (according to Table S1) and duplicate doubling dilutions were performed in dilution buffer for each serum sample. Plates were incubated for 1 hour at 37 °C and washed 3 times with NaCl-Brij-NaN_3_ buffer. Next, 100 µL appropriate alkaline phosphatase conjugated detection antibody was added to each well, incubated for 1 hour at 37 °C and washed 3 times with NaCl-Brij-NaN3 buffer. Wells were incubated for 1 hour at room temperature in the dark with 100 μL *p*-Nitrophenyl Phosphate liquid substrate (Sigma, N7653) then reactions stopped with 50 µL 3 M NaOH stop solution. Absorbance readings were measured at 405 nm using a kinetic microplate reader (Molecular Devices) and SoftMax Pro software. The mean of the duplicates was determined. From six assays using human and sheep antisera, the PLL ELISA assay had a % GCV of 4% and 6%, respectively.

*S.1.5 HPAEC-PAD*

Saccharide content of pre-treated Vi was determined by High-Performance Anion-Exchange Chromatography with Pulsed Amperometric Detection (HPAEC-PAD), using a Dionex (Thermo Scientific) ICS-5000 chromatography system with an Amino Trap guard column and a CarboPac PA1 analytical column (2 mm x 250 mm). Samples were diluted to approximately 20 µg/mL and quantitative standard of 0.5 to 27 µg/mL *C. freundii* Vi at 5 levels was subjected to alkaline hydrolysis with 2 M NaOH at 110 °C for 4 hours, in a heating block [4]. An identical amount of *N*-acetyl-D-glucosamine-6-phosphate (Sigma 92689) was added to all samples and standards as internal spike to harmonize detector variation. Standards were injected in triplicate throughout the run, and samples were injected twice in succession. Sample volume of 15 µL were injected using a 50 µL loop, then eluted with multistep gradient as: 0-2 min, 100 mM NaOH, 40 mM NaNO_3_; 2-22 min, 100 mM NaOH, 40-150 mM NaNO_3_; 22-31 min, 100 mM NaOH, 40 mM NaNO_3_, with flow rate of 0.25 ml/min. Peaks were detected using a quadruple potential waveform for carbohydrates: E1 = 0.1 V, t1 = 400 ms; E2 = -2 V, t2 = 20 ms; E3 = 0.6 V, t3 = 10 ms; E4 = -0.1 V, t4 = 60 ms. Chromeleon software (Version 7.2) was used to program the run and analyze data. Assay precision was determined to be 4.3%, using the median of % CV from this laboratory’s performance in a collaborative study [5].

*S.1.6 SEC-MALS-Intrinsic Viscometry*

A High-Performance Liquid Chromatography - Size Exclusion Chromatography (HPLC-SEC, ICS5000^+^, Dionex, Thermo Scientific) System with a Refractive Index detector (RI, Optilab T-rEX, Wyatt Technology), Multi Angle Laser Light Scattering detector (MALS, DAWN HELEOS-II, Wyatt Technology) and a Viscometer detector (ViscoStar-II, Wyatt Technology) was used for analysis. A polymer-based column, TSKgel G5000PW_XL_ analytical column (Tosoh Bioscience GmbH) with a fractionation range of MW 4000 to 1x10^6^, with a TSKgel PW_XL_ guard column was used for the determination. System suitability tests were performed using DNA, thyroglobulin, bovine serum albumin, carbonic anhydrase and tyrosine. BSA standard (Pierce 23209) was used to normalize the MALS detector, and to determine the inter-detector delay. BSA was run at the beginning of an experiment set and mass values were within 5% of theoretical mass. The following eluents were used: 20 mM Hepes (Sigma H3375), pH 7.0-7.4, PBS, pH 7.3-7.5, or water.

PSs were diluted to 500 µg/mL using eluent. A sample of 100 µL (50 µg of PS) was loaded onto the column for chromatography with flow rate of 0.3 or 0.6 mL/min. Samples were injected in duplicate or triplicate and the eluted PS was monitored by refractive index, together with signals from the MALS and viscometer detectors.

Chromeleon software version 7.2 (Thermo Scientific) was used to program the run on HPLC-SEC. Astra software version 6.1 (Wyatt Technology) was used for data analysis. The % mass recovery is determined using the ratio of the measured amount recovered from the refractive index signal, using a constant refractive index increment (dn/dc) of 0.14 mL/g for polysaccharides, to the known amount loaded, as determined in WHO Collaborative Studies. Data from the RI detector, and MALS detectors arranged at 44.8, 53.1, 61.1, 70.1, 80.1, 90.0, 99.9, 109.9, 120.1, 130.4 and 140.0 degrees to the flow-path were used for determination of the weight-average molecular mass (Mw, g/mol) and polydispersity (Mw/Mn) using Zimm formalism. Data from the viscometer and the light scattering detectors were used to determine the hydrodynamic radius moments (Rh(v), nm) and intrinsic viscosity moments ([η]n, mL/g) using the Huggins formalism. Instrument errors determined by Astra software were ≤ 6% for mass.

**S.2.0. Results**

*S.2.1. Effect of de-O-acetylation on antibody binding*

Multi-dilution antibody binding curves reported in the main Results section 3.2 from the mHSA ELISA (Fig S1) and Capture ELISA (Fig S2) are included.


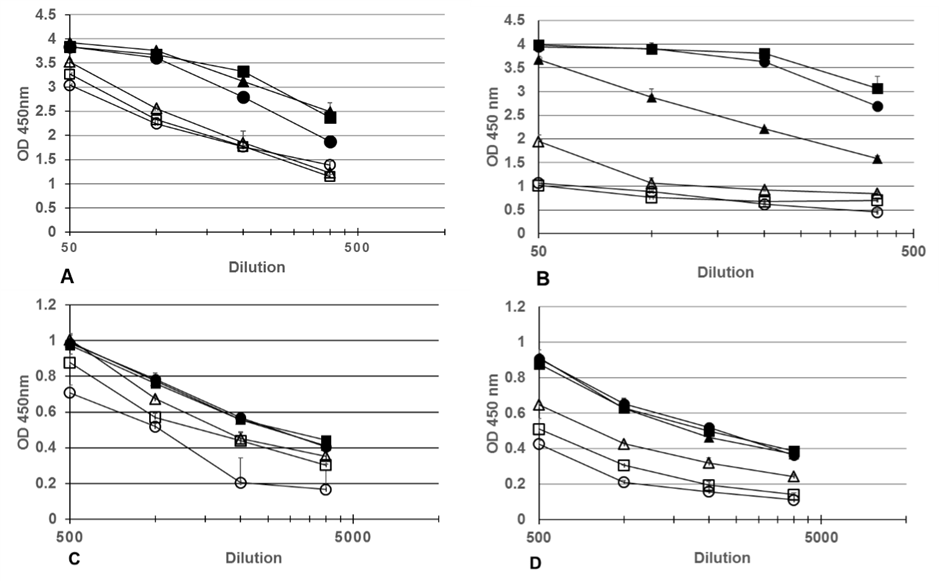


**Fig. S1*.*** Effect of de-*O*-acetylation of *C. freundii* [A, B] and *S. typhi* Vi from [C, D] on binding to polyclonal human [A,C] and sheep anti-Vi antibody [B,D] from indirect mHSA ELISA. Data points are the average of two assays, with untreated PS (●), or following de-*O*-acetylation with 0.05 M (⏹), 0.3 M (▲), 0.7 M (△), 1.0 M (□) or 1.5 M (🔾) ammonium hydroxide.


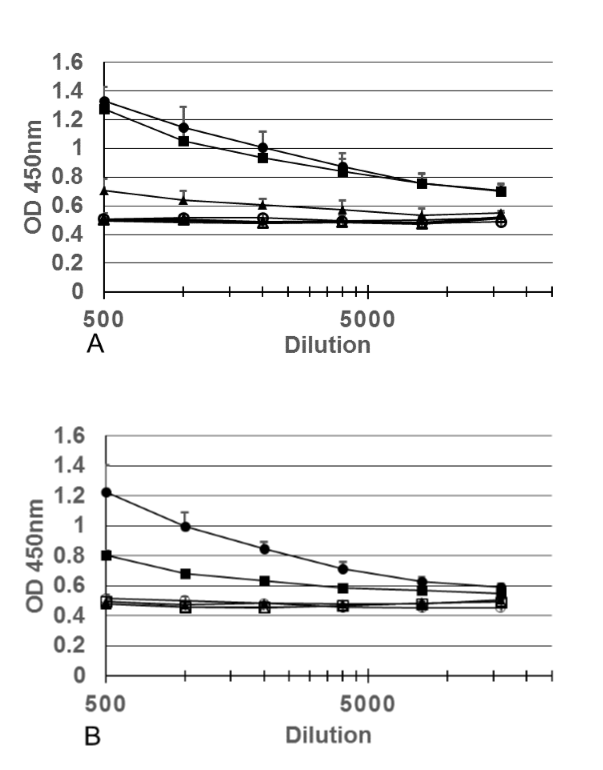


**Fig. S2.** Effect of de-*O*-acetylation of Vi samples from *C. freundii* and *S.* Typhi on binding to mouse monoclonal anti-Vi antibody used as capture antibody. *C. freundii* (A) and *S.* Typhi (B) binding curves contain the same symbols as used for Fig. S1. Data points are the mean of two assays (n=4).

*S.2.2. Further characterization of C. freundii and S. Typhi Vi standards*

The HPLC SEC chromatograms as reported in the main Results section 3.3 are shown in Fig S3.


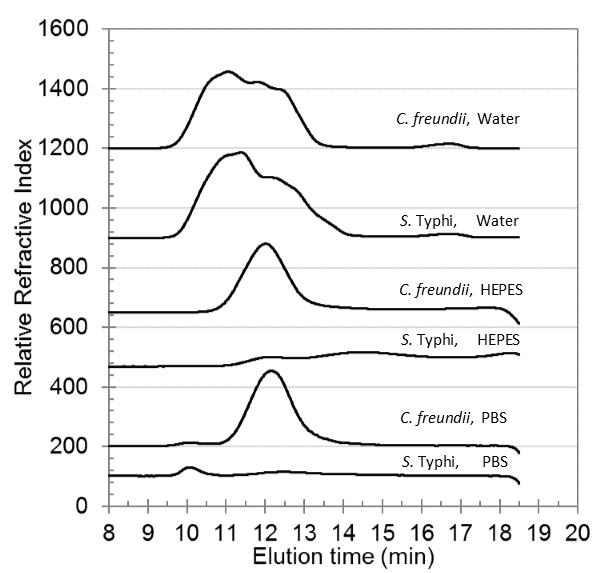


**Fig. S3.** Sizing chromatograms of Vi standards from TSKgel G5000PW_XL_ column in different eluents. The column void marker (DNA) eluted at 9.0 min, and the total column volume marker (tyrosine) at 20.9 min in water and in PBS, pH 7.3-7.5, and at 9.9 min and at 21.9 min in Hepes buffer, pH 7.0-7.4.

**
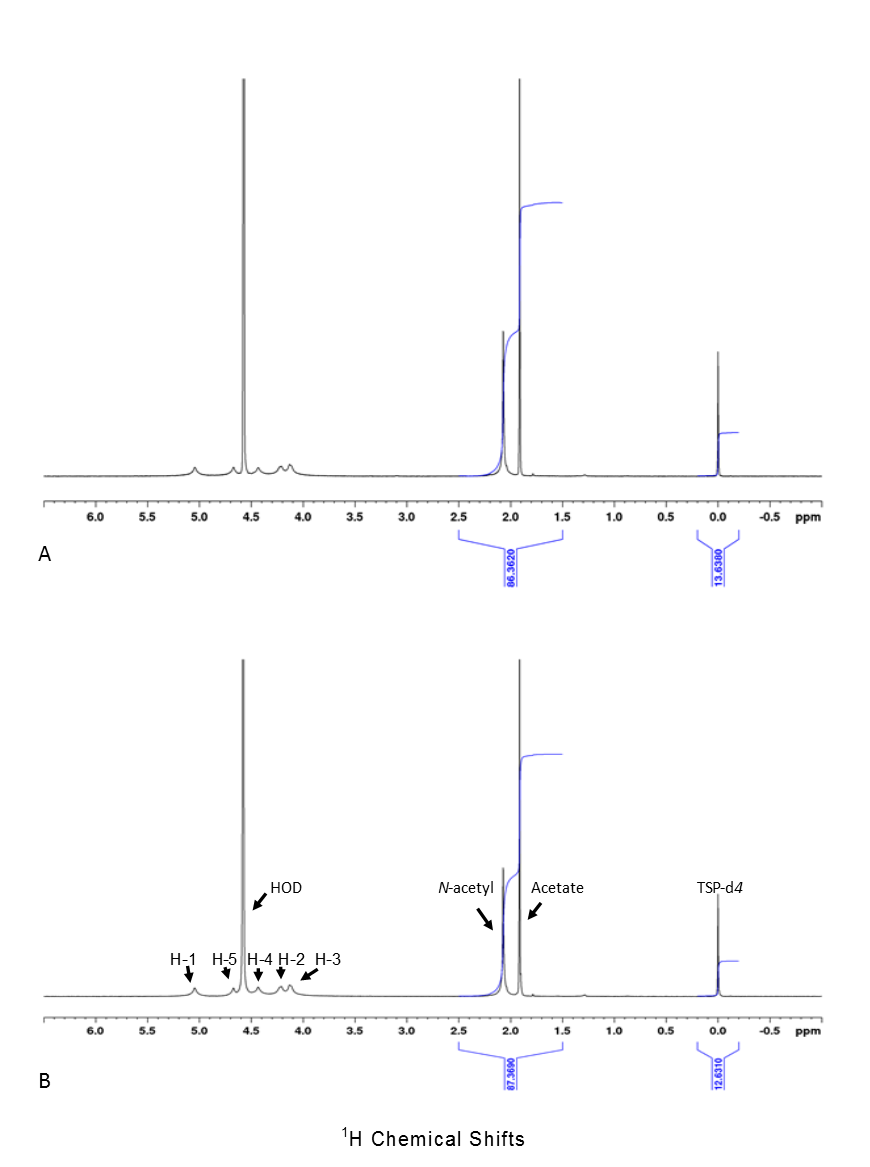
**

**Fig S4.** ^1^H-NMR spectra of Vi standards from *C. freundii* (A) and *S.* Typhi (B). Vi standards were reconstituted at 1.4 mg/mL in deuterium oxide containing 0.2 M sodium deuteroxide and 0.128 mg/mL sodium trimethylsilyl-2,2,3,3-^1^H-propionate. Data are reproduced from the NIBSC report to ECBS collaborative study 574 [5]. Vi PS peak assignment are labelled and the integral regions from *N*-acetyl (2.07 ppm), acetate (1.910 ppm) and TSP-*d4* (0 ppm) shown.

**S.3.0. References**

[1] Rijpkema S, Hockley J, Logan A, Rigsby P, Atkinson E, Jin C, Goldblatt D, Liang H, Bachtias NS, Yang JS, Goel A, Ramasamy V, Pasetti MF, Pollard AJ, the anti-Vi IgG working group. Establishment of the first international standard for human anti-typhoid capsular Vi polysaccharide IgG. Biologicals 2018;56:29-38. <https://doi.org/10.1016/j.biologicals.2018.09.001>

[2] Lin FY, Ho VA, Khiem HB, Trach DD, Bay PV, Thanh TC, Kassaczka Z, Bryla DA, Shiloach J, Robbins JB, Schneerson R, Szu SC. The efficacy of a *Salmonella* typhi Vi conjugate vaccine in two-to-five- year-old Children. N Engl J Med 2001;344(17):1263-9. <https://www.nejm.org/doi/full/10.1056/NEJM200104263441701>

[3] Arakere G, Frasch CE. Specificity of antibodies to *O*-acetyl-positive and *O*-acetyl-negative group C Meningococcal polysaccharides in sera from vaccinees and carriers. Infect Immun 1991;59(12):4349-4356. PMID: [1937795](https://www.ncbi.nlm.nih.gov/pubmed/1937795) PMCID: [PMC259048](https://www.ncbi.nlm.nih.gov/pubmed/1937795)

[4] Micoli F, Rondini S, Pisoni I, Proietti D, Berti F, Costantino P, Rappuoli R, Szu S, Saul A, Martin LB. Vi-CRM_197_ as a new conjugate vaccine against *Salmonella* Typhi. Vaccine 2011;29(4):712-720. <https://doi.org/10.1016/j.vaccine.2010.11.022>

[5] WHO. Evaluation of candidate International Standards for Vi polysaccharide from *Citrobacter freundii* and *Salmonella enterica* subspecies *enterica* serovar Typhi. In: Expert Committee on Biological Standardization, Geneva, 17 to 20 October 2017 (WHO/BS/2017.2310). <http://www.who.int/biologicals/expert_committee/BS2310_Vi_PS_Report_for_WHO_Final.pdf?ua=1> Accessed 22 March, 2017.
